# ^177^Lu-labelled peptide receptor radionuclide therapy in patients with neuroendocrine tumors: a systematic review and meta-analysis

**DOI:** 10.3389/fendo.2026.1758639

**Published:** 2026-02-19

**Authors:** Jingrong Wang, Xangyi Pang, Jie Lian, Haibo Lu

**Affiliations:** 1 Department of Outpatient Chemotherapy, Harbin Medical University Cancer Hospital, Harbin, Heilongjiang, China; 2 Heilongjiang Province Key Laboratory of Research on Molecular Targeted Anti-Tumor Drugs, Harbin, China

**Keywords:** 177Lu-DOTATATE, gastroenteropancreatic NETs, meta-analysis, neuroendocrine tumor, peptide receptor radionuclide therapy

## Abstract

**Objective:**

This systematic review and meta-analysis evaluated the efficacy and safety of ^177^Lu-labelled peptide receptor radionuclide therapy (PRRT) in neuroendocrine tumors (NETs).

**Methods:**

This systematic review and meta-analysis conducted a search of the PubMed/MEDLINE, Embase, and Web of Science databases. Included studies assessed treatment outcomes using Response Evaluation Criteria in Solid Tumors (RECIST) or World Health Organization (WHO) criteria. A random-effects model was used to calculate pooled proportions.

**Results:**

A total of 14 studies with 1844 patients were included. The pooled disease control rate (DCR) was 87.6% (95% Confidence Interval (CI), 82.5%-92%), and the objective response rate (ORR) was 32.2% (95% CI, 25.4%-39.3%). Median progression-free survival (PFS) was 30.87 months (95% CI, 22.71–39.04), and median overall survival (OS) was 51.85 months (95% CI, 39.99–63.71). Subgroup analysis revealed a significantly higher DCR in grade 1–2 NETs 97.7% (95% CI, 91.4%-100%) compared to grade 3 NETs 90.8% (95% CI, 85.1%-94.4%), and a higher ORR in grade 1–2 tumors 45.4% (95% CI, 35.3%-55.6%) compared to grade 3 tumors 27.1% (95% CI, 21.2%-33.4%). PFS was longer in pancreatic NETs 93.9 months (95% CI, 39.45–148.35) than in gastrointestinal NETs 66.32 months (95% CI, 41.78–90.87). The overall incidence of adverse events was 4.1%, with grade ≥3 toxicities in 4.3%. ^177^Lu-PRRT demonstrates high efficacy and a favorable safety profile in treating NETs.

**Conclusion:**

^177^Lu-DOTA-Tyr3-octreotate (^177^Lu-DOTATATE) demonstrates high efficacy and a favorable safety profile in treating NETs.

**Systematic Review Registration:**

https://www.crd.york.ac.uk/prospero/, identifier CRD420251047030.

## 1 Introduction

Neuroendocrine tumors (NETs) are malignant neoplasms arising from neuroendocrine cells, most commonly occurring in the digestive system. Their incidence has increased significantly, from 1.09 per 100,000 in 1973 to 6.98 per 100,000 in 2012, with current rates ranging from 5.25 to 7 per 100,000, according to Surveillance, Epidemiology, and End Results data (1).

First-line treatment strategies for metastatic or unresectable NETs typically include chemotherapy and somatostatin analogs. However, conventional therapies have limitations in managing disease progression. Recent advancements in peptide receptor radionuclide therapy (PRRT) have proven effective, particularly for well-differentiated G1–G2 gastroenteropancreatic NETs (GEP-NETs) (2).PRRT, using radionuclide-coupled analogs like ^177^Lu-DOTA-Tyr3-octreotate (^177^Lu-DOTATATE) and ^90^Y-DOTA-Tyr³-octreotide (^90^Y-DOTATOC), targets tumor cells. ^177^Lu-DOTATATE, with fewer adverse effects, has Food and Drug Administration and European Medicines Agency approval for receptor-positive GEP-NETs in adults. The recommended regimen is four cycles of 7.4 GBq (200 mCi) (3–5). The NETTER-1 trial demonstrated superior progression-free survival (PFS) and objective response rate (ORR) in the ^177^Lu-DOTATATE group compared to the control (6). Despite these promising clinical outcomes, safety considerations remain paramount and comprehensive efficacy data across heterogeneous NET subgroups require further elucidation.

Safety concerns primarily involve reversible hematologic toxicity and nephrotoxicity, with common adverse effects including anemia, leukopenia, and thrombocytopenia (7). Few meta-analysis have assessed the efficacy of ^177^Lu-DOTATATE in different NET subgroups. This meta-analysis retrospectively evaluated the efficacy and safety of ^177^Lu-DOTATATE across various NET grades and patient subgroups.

## 2 Methods

### 2.1 Literature search strategy

This meta-analysis was conducted in strict accordance with the Preferred Reporting Items for Systematic Reviews and Meta-Analyses guidelines (8). We systematically searched the PubMed/MEDLINE, Embase, and Web of Science databases for relevant literature up to April 8, 2025. Search terms included “neuroendocrine tumor”, “pancreatic neuroendocrine tumor”, “gastroenteropancreatic neuroendocrine tumor”, “PRRT”, and “^177^Lu-DOTATATE”, along with related variants of these terms. Additionally, we manually searched the reference lists of relevant articles to identify any further relevant studies.

### 2.2 Eligibility criteria

Inclusion Criteria:

- Patients with histologically confirmed NETs.
- Treatment with ^177^Lu-DOTATATE as the sole therapeutic modality (monotherapy).
- Evaluation of treatment response according to the Response Evaluation Criteria in Solid Tumors (RECIST) or World Health Organization (WHO) criteria.

Exclusion Criteria:

- Publications that are review articles, conference abstracts, editorials, or case reports.
- Preclinical (animal) studies.
- Studies lacking sufficient raw data for extraction or analysis of treatment outcomes.

### 2.3 Quality assessment

The risk of bias (RoB) assessment was performed using the latest Cochrane Collaboration’s RoB tool for randomized controlled trials (RCTs) (9). This tool evaluates six domains: randomization process, deviations from intended interventions, missing outcome data, outcome measurement, selective reporting, and overall RoB, providing an overall judgment (9). For cohort studies and case-control studies, the Risk of Bias in Nonrandomized Studies of Interventions (ROBINS-I) tool was used for analysis (10). The overall risk of bias for each study was classified as low, moderate, serious, or critical.

### 2.4 Data extraction

Two researchers independently extracted data from the included studies, collecting the following information: first author, year of publication, study design, number of patients, tumor type, average therapeutic dose of radiopharmaceuticals, treatment duration, and follow-up period. The primary outcomes were efficacy and adverse events. Efficacy outcomes included disease control rate (DCR), ORR, pooled PFS, and overall survival (OS). DCR was defined as the percentage of patients achieving complete response, partial response, or stable disease, and ORR as the percentage achieving complete response or partial response. PFS was defined as the time from treatment initiation to tumor progression (e.g., enlargement or metastasis) or death, and OS as the time from treatment initiation to death from any cause. Imaging responses were assessed according to RECIST and mRECIST criteria (11, 12). Adverse events were recorded in accordance with the National Cancer Institute Common Terminology Criteria for Adverse Events version 3.0, with an emphasis on hematologic and renal toxicities (13).

### 2.5 Statistical analysis

A meta-analysis was performed using Stata version 18.0 (StataCorp LLC, College Station, TX, USA) and R version 3.5.2 (R Foundation for Statistical Computing, Vienna, Austria). The efficacy of ^177^Lu-DOTATATE treatment was assessed by calculating the DCR, ORR, PFS, and OS, each reported with 95% confidence intervals (CI). Treatment-related adverse events were also evaluated and reported with 95% CI. Statistical heterogeneity among studies was assessed using Cochran’s Q test and the Higgins I² statistic. Heterogeneity was considered significant if the Cochran’s Q test *P*-value was < 0.10 and I² was > 50%; in this case, a random-effects model was applied (14). Otherwise, a fixed-effects model was used. Sensitivity analyses were conducted by sequentially excluding each study (leave-one-out analysis) to test the robustness of the results. Statistical significance was defined as *P* < 0.05.

## 3 Results

### 3.1 Study selection

**Figure 1:**
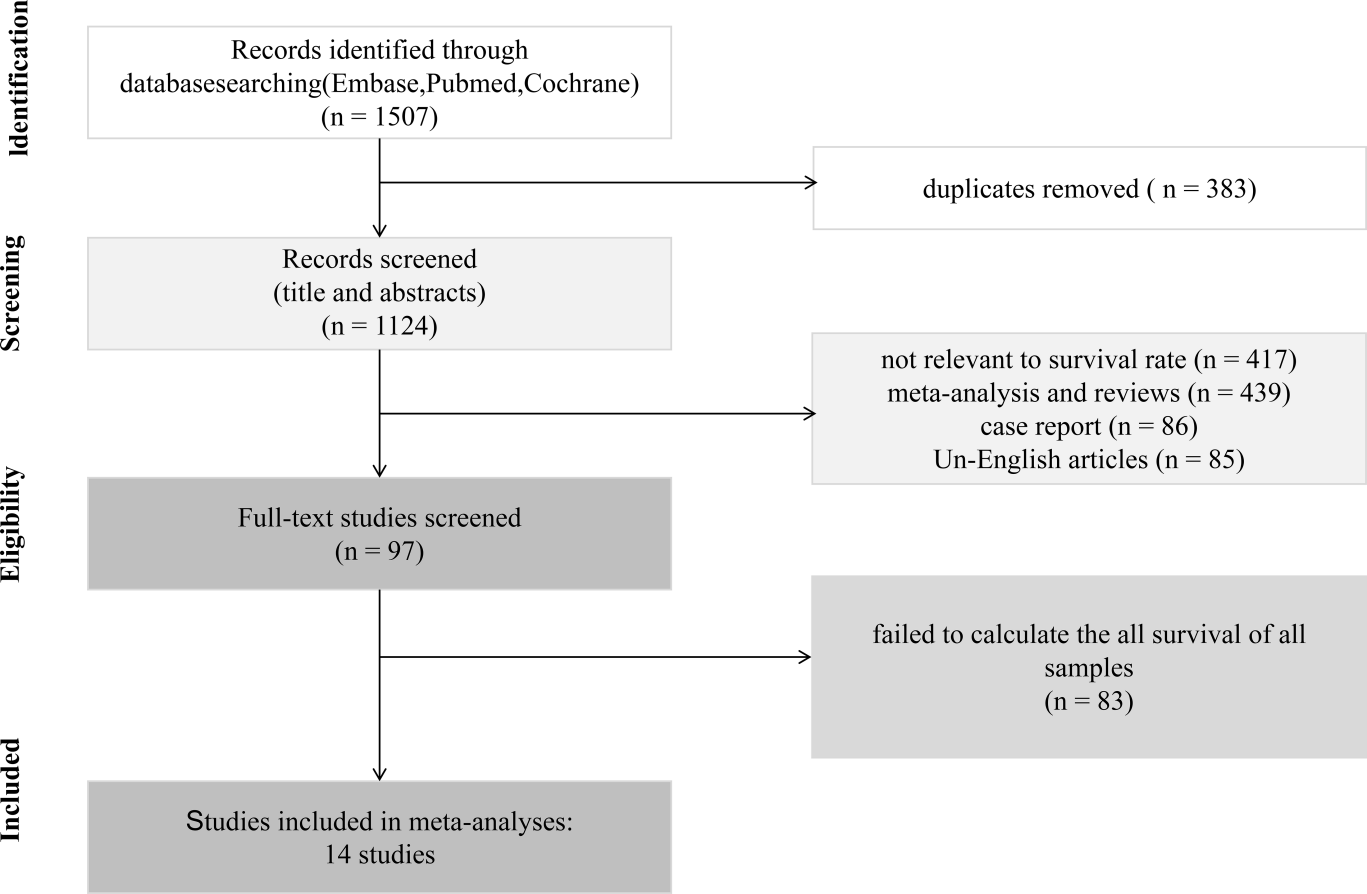
Flow diagram of the study selection.

Systematic searches of electronic databases identified 1507 records (**Figure 1**). After removing 383 duplicate records, we screened the titles and abstracts of the remaining 1124 articles. During the title/abstract screening, 1,027 records were excluded due to being irrelevant to survival rates, meta-analyses and review articles, case reports, and non-English publications. A total of 97 articles were retained for full-text review. Full-text assessment led to the exclusion of 83 articles, primarily due to inappropriate publication type or failure to report DCR or ORR. Ultimately, 14 studies met all inclusion criteria and were included in the meta-analysis.

### 3.2 Study characteristics

The 14 included studies comprised 5 retrospective and 9 prospective investigations; 12 employed a cohort design, and 2 were RCTs (**Table 1**). Cohort studies were assessed using the ROBINS-I, and RCTs were evaluated with the Cochrane Collaboration’s RoB tool. All included studies were judged to have a low overall RoB, and the three RCTs were considered to be of high methodological quality. We enrolled a total of 1844 patients. The baseline characteristics of the study participants are presented in **Table 2**.

**Table 1:** Methodological quality or risk of bias assessment of the included studies.

First author	Year	Study type	Study design	Follow-up months median (Range)	Quality
Partelli (15)	2024	Prospective	Cohort	4	Serious risk
Akhavanallaf (16)	2024	Retrospective	Cohort	23.9 (19.3-32.4)	Serious risk
Delpassand (17)	2024	Retrospective	Cohort	39.6 (30-54)	Serious risk
Singh (NETTER-2) (18)	2024	Prospective	RCT	23.2 (16.4 - 28.8)	Low risk
Kennedy (19)	2022	Retrospective	Cohort	68	Moderate risk
Minczeles (20)	2022	Retrospective	Cohort	34 (28-40)	Moderate risk
Mitjavila (21)	2022	Retrospective	Cohort	21.2	Serious risk
Parghane (22)	2021	Prospective	Cohort	24 (12-36)	Serious risk
Ortega (23)	2021	Prospective	Cohort	12.2	Serious risk
Strosberg (NETTER-1) (6)	2021	Prospective	RCT	76.3 (0.4-95.0)	Low risk
Paganelli (24)	2020	Prospective	Cohort	118 (12.6-139.6)	Serious risk
Braat (25)	2020	Prospective	Cohort	6	Moderate risk
Reidy-Lagunes (26)	2019	Prospective	Cohort	32	Serious risk
Van der Zwan (27)	2018	Prospective	Cohort	88.6 (79-98.2)	Moderate risk

**Table 2:** Baseline characteristics of the included articles.

First author	Year	Country	No. patients	Activity, GBq	177Lu cycles	Tumor location	Response criteria	DCR	ORR
Partelli	2024	Italy	31	7.4	4	Pancreatic NETs	RECIST1.1	1	0.581
Akhavanallaf	2024	USA	91	7.4	4	GEP-NETs	68Ga-DOTATATE PET/CT	–	–
Delpassand	2024	USA	31	–	6	GEP-NETs	RECIST1.1	1	0.355
Singh (NETTER-2)	2024	USA	261	7.2 - 7.5	4	GEP-NETs	RECIST1.1	0.907	0.43
Kennedy	2022	Italy	104	7.8	1-11	GEP-NETs	RECIST1.1	0.929	0.238
Minczeles	2022	NL	243	7.4	3-4	GEP-NETs	Unkown	0.746	0.3
Mitjavila	2022	ES	417	7.4	4	GEP and bronchopulmonary NETs	RECIST1.1	0.861	0.348
Parghane	2021	India	57	7.4	4-5	GEP-NETs	RECIST1.1 PERCIST 68Ga-DOTATATE PET/CT	0.93	0.404
Ortega	2021	Italy	91	7.4	3.6	GEP and bronchopulmonary NETs	68Ga-DOTATATE PET/CT	0.78	0.22
Strosberg (NETTER-1)	2021	USA	231	7.4	4	Midgut NETs	RECIST1.1	0.829	0.18
Paganelli	2020	Italy	43	3.7/5.5	5	GI NETs	WHO	–	–
Braat	2020	NL	30	7.4	4	GEP and bronchopulmonary NETs	RECIST1.1	0.833	0.433
Reidy-Lagunes	2019	USA	20	3.7/7.4	1-2	GEP and bronchopulmonary NETs	RECIST1.1	0.85	0.45
Van der Zwan	2018	NL	194	–	2	GEP and bronchopulmonary NETs	RECIST1.1	0.75	0.155

### 3.3 Disease control rates

**Figure 2:**
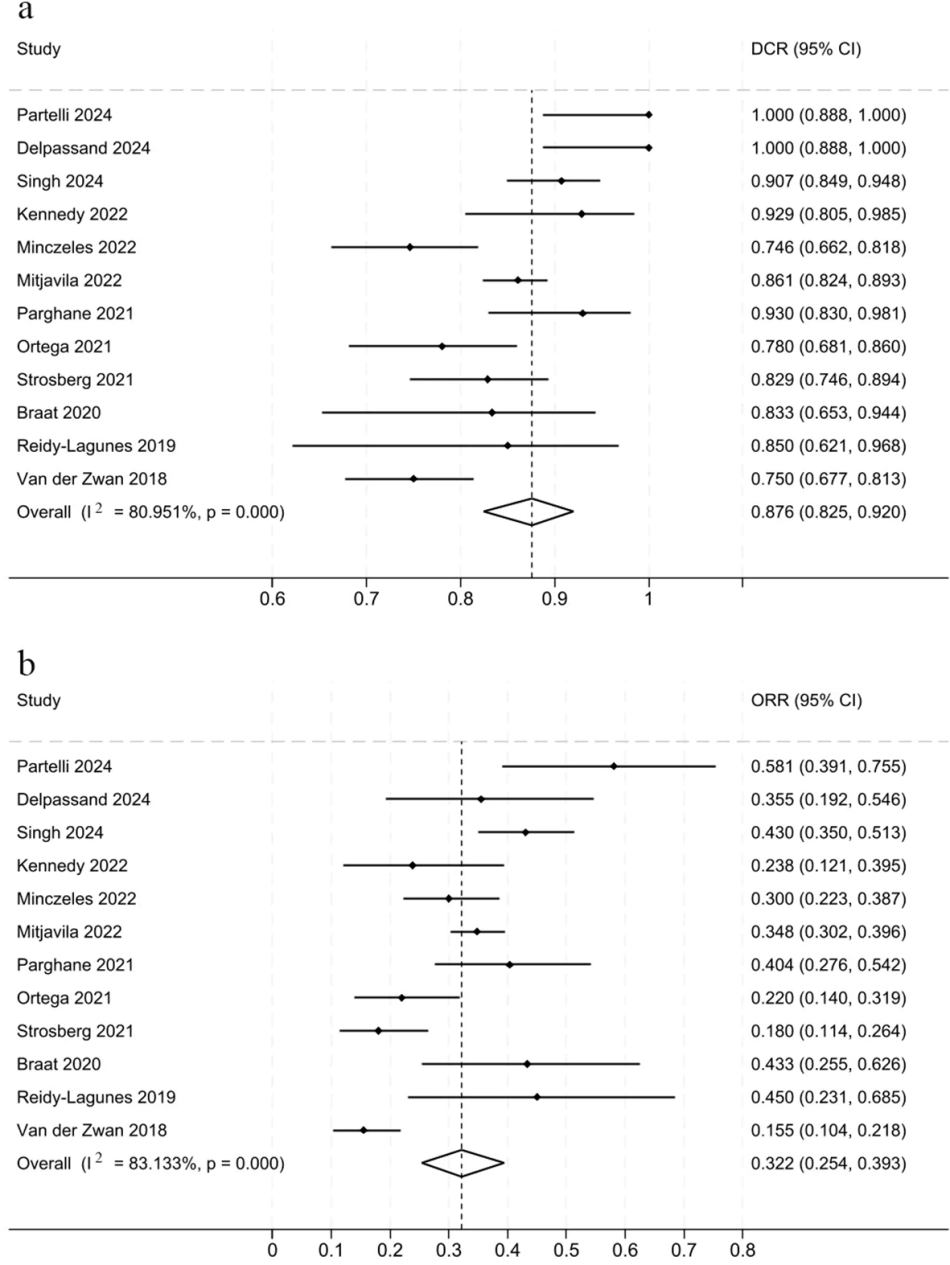
Forest plot of odds ratios summarizing the efficacy of ^177^Lu-DOTATATE in patients with NETs. DCR **(a)** and ORR **(b)**. The horizontal lines denote the upper and lower bounds of the 95% CI. *P*-value greater than 0.05 are considered to indicate statistical non-significance. ^177^Lu-DOTATATE - ^177^Lu-DOTA-Tyr3-octreotate; NETs - neuroendocrine tumors; DCR - disease control rate; ORR - objective response rates; CI - confidence intervals.

We analyzed the DCR from 12 studies involving a total of 1279 patients (**Figure 2a**). The Cochran Q test yielded a *P*-value of <0.05 and an I² of 80.95%, indicating significant heterogeneity among the studies. Therefore, a random-effects model was employed for the meta-analysis. The DCR across studies ranged from 74.6% to 100%. The pooled data from all studies revealed a pooled DCR of 87.6% (95% CI, 82.5%-92%) for ^177^Lu-DOTATATE treatment in NETs. The sensitivity analysis revealed consistent effect size estimates, ranging from 0.8546 to 0.9054, following the sequential exclusion of individual studies (**Supplementary Figure 1**) (**Supplementary Table 1**). The combined effect rate of the remaining studies was 86%, indicating that the results were stable and reliable.

### 3.4 Objective response rate

In addition to evaluating disease control, we further analyzed the ORR. A total of 12 studies involving 1279 patients were included in the analysis (**Figure 2b**). The results, derived from a random-effects model, demonstrated that the pooled ORR of ^177^Lu-DOTATATE in NET-treated patients was 32.2% (95% CI, 25.4%-39.3%). The study included 30 patients, all with G1 or G2 grade NETs (15).This study reported a notably high ORR of 58.1%, which may be attributable to the small sample size and the specific NETs grading, which differed from other studies.

### 3.5 Progression-free survival

**Figure 3:**
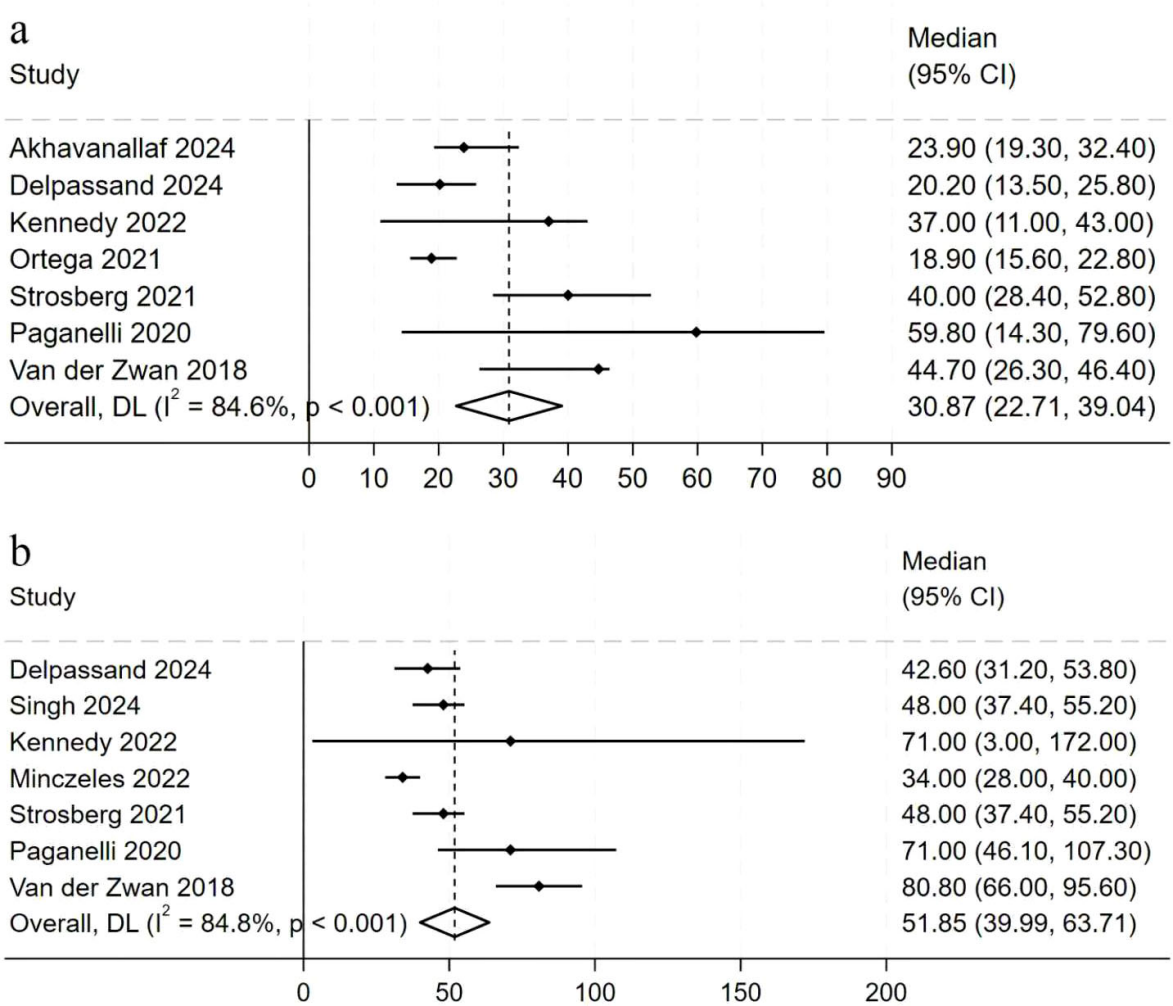
Forest plot of hazard ratios summarizing the efficacy of ^177^Lu-DOTATATE in patients with NETs. PFS **(a)** and OS **(b)**. The horizontal lines denote the upper and lower bounds of the 95% CI. *P*-value greater than 0.05 are considered to indicate statistical non-significance. ^177^Lu-DOTATATE - ^177^Lu-DOTA-Tyr3-octreotate; NETs - neuroendocrine tumors; PFS - progression-free survival; OS - overall survival; CI - confidence intervals.

PFS is a critical measure of the long-term benefits of treatment. We analyzed the PFS data from seven studies involving 665 patients (**Figure 3a**). Seven additional studies were excluded due to missing data. The meta-analysis using the random-effects model showed a pooled PFS of 30.87 months (95% CI, 22.71-39.04 months), with individual study PFS ranging from 18.9 to 59.8 months. The study reported the longest PFS at 59.8 months, though this study had the smallest sample size and the widest 95% confidence interval, indicating greater uncertainty in the result (24). The results of the sensitivity analysis confirmed the robustness of the combined PFS estimate (**Supplementary Figure 2**) (**Supplementary Table 2**). Sequential exclusion of individual studies maintained the PFS of the remaining studies at approximately 28.34 months, with no significant changes observed. This demonstrates the robustness and reliability of the combined PFS results.

### 3.6 Overall survival

In evaluating long-term efficacy, we examined OS after ^177^Lu-DOTATATE therapy in NETs. The figure illustrates OS data from seven clinical studies involving 676 patients (**Figure 3b**). Using a random-effects model, the pooled OS was 51.85 months (95% CI, 39.99–63.71), with a range of 34 to 80.8 months.

### 3.7 Subgroup analysis

**Figure 4:**
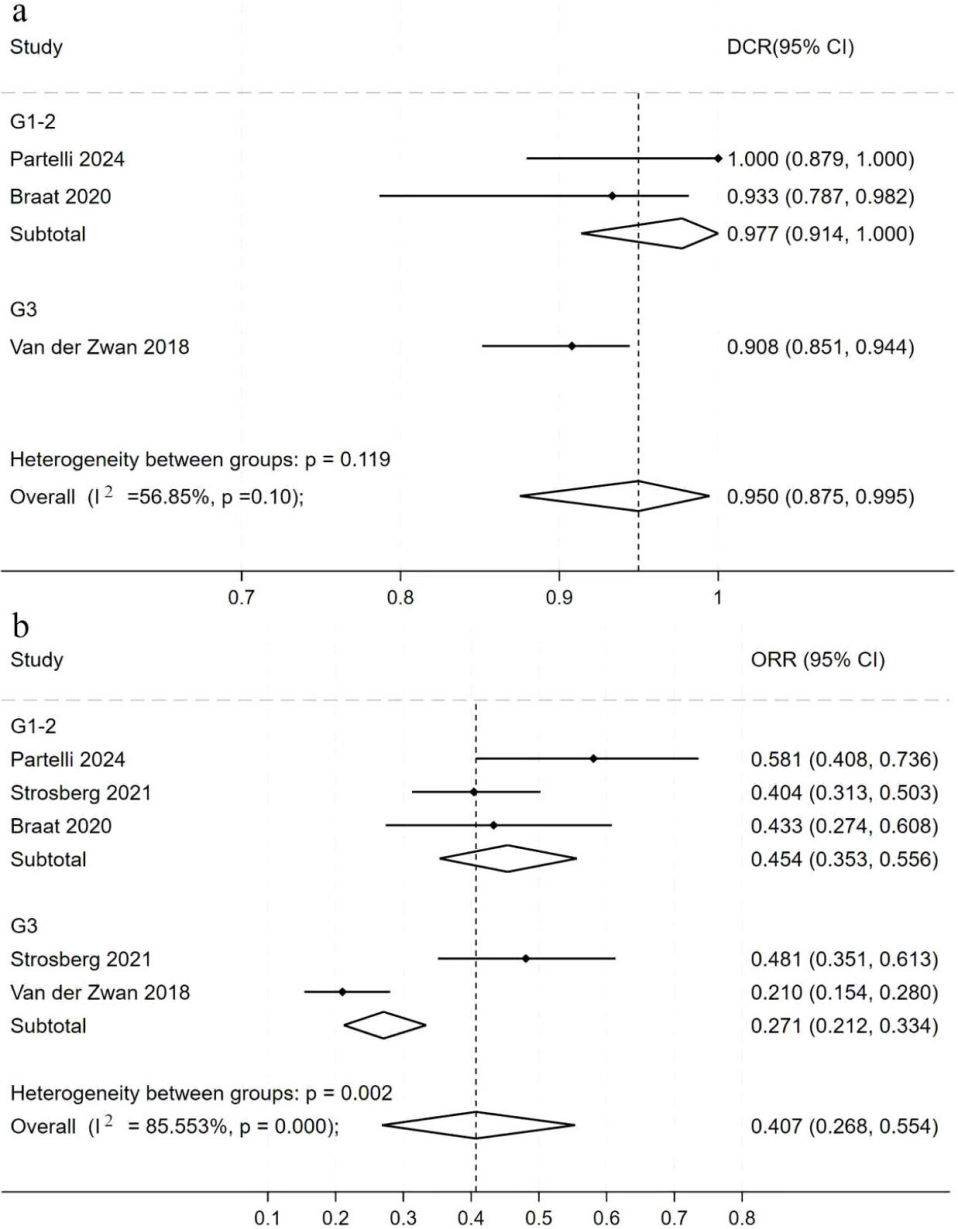
Forest plot of odds ratios comparing ^177^Lu-DOTATATE efficacy across tumor grade subgroups in patients with NETs. **(a)** DCR; **(b)** ORR. The horizontal lines denote the upper and lower bounds of the 95% CI. *P*-value greater than 0.05 are considered to indicate statistical non-significance. ^177^Lu-DOTATATE - ^177^Lu-DOTA-Tyr3-octreotate; NETs - neuroendocrine tumors; DCR - disease control rate; ORR - objective response rates; CI - confidence intervals.

Due to observed heterogeneity in efficacy outcomes, subgroup analysis were performed to explore potential factors. First, DCR and ORR were compared between G1–2 and G3 NETs; next, PFS was compared across tumor locations. We first analyzed DCR by grade (**Figure 4a**). For G1–2 NETs (2 studies, 58 patients), the pooled DCR was 97.7% (95% CI, 91.4%–100%). For G3 NETs (152 patients), the pooled DCR was 90.8% (95% CI, 85.1%–94.4%). The DCR for G1–2 tumors was slightly higher than for G3 tumors. However, the between-group *P*-value was 0.119 (*P* > 0.05), indicating no significant difference in DCR between the grades. Given heterogeneity in DCR and ORR across studies, we further analyzed ORR by grade (**Figure 4b**). The between-group *P*-value was 0.002 (*P* < 0.05), indicating significant heterogeneity. For G1–2 NETs (3 studies, 160 patients), the pooled ORR was 45.4% (95% CI, 35.3%–55.6%). For G3 NETs (2 studies, 209 patients), the pooled ORR was 27.1% (95% CI, 21.2%–33.4%). Thus, the ORR for G3 tumors was significantly lower than that for G1–2 tumors.

**Figure 5:**
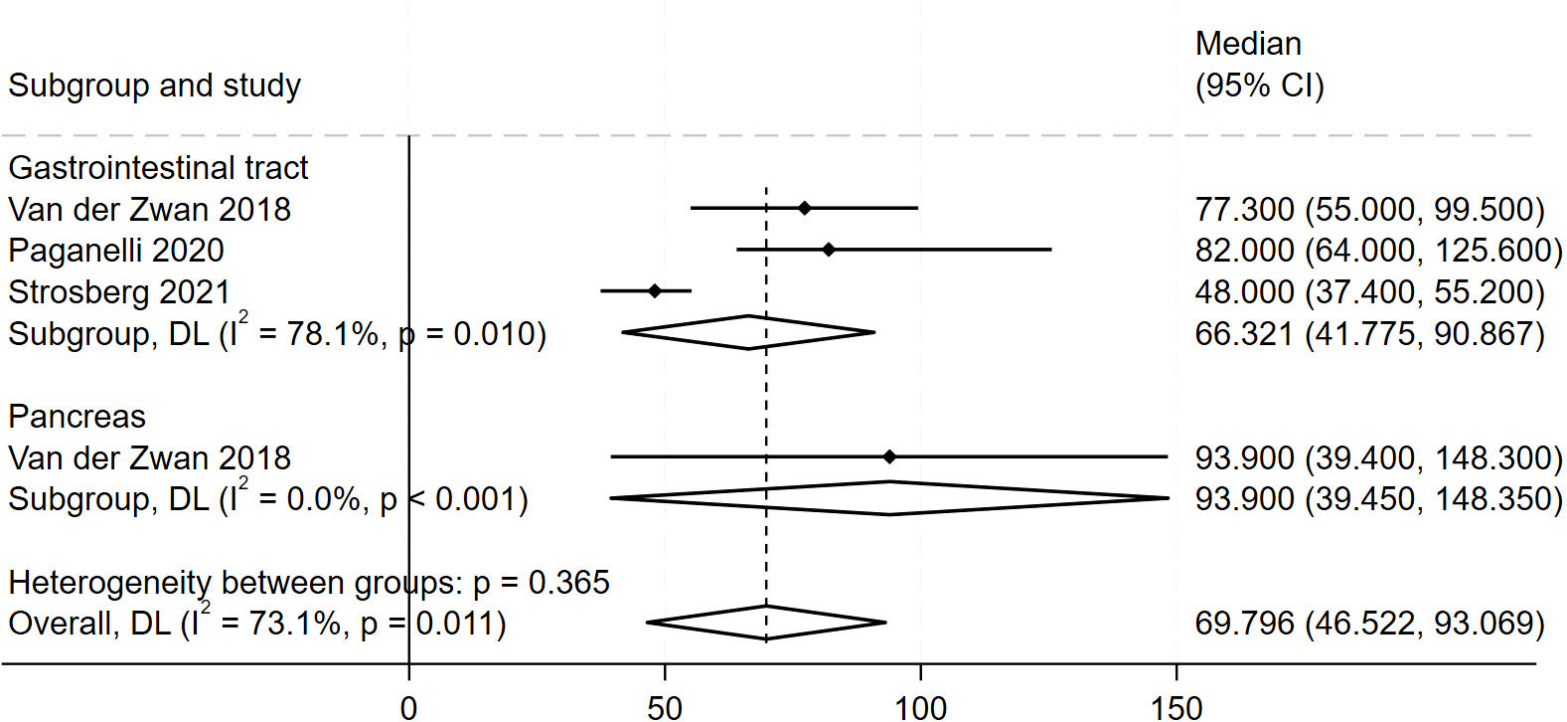
Forest plot of odds ratios comparing PFS across tumor site subgroups in patients treated with ^177^Lu-DOTATATE. The horizontal lines denote the upper and lower bounds of the 95% CI. *P*-value greater than 0.05 are considered to indicate statistical non-significance. ^177^Lu-DOTATATE - ^177^Lu-DOTA-Tyr3-octreotate. NETs - neuroendocrine tumors; PFS - progression free survival; CI - confidence intervals.

We also performed a subgroup analysis by tumor location (**Figure 5**). Four studies involved gastrointestinal NETs; the pooled PFS was 66.32 months (95% CI, 41.78–90.87). One study involved pancreatic NETs, reporting a PFS of 93.9 months (95% CI, 39.45–148.35). Patients with pancreatic NETs had significantly better PFS than those with gastrointestinal NETs.

### 3.8 Safety

**Figure 6:**
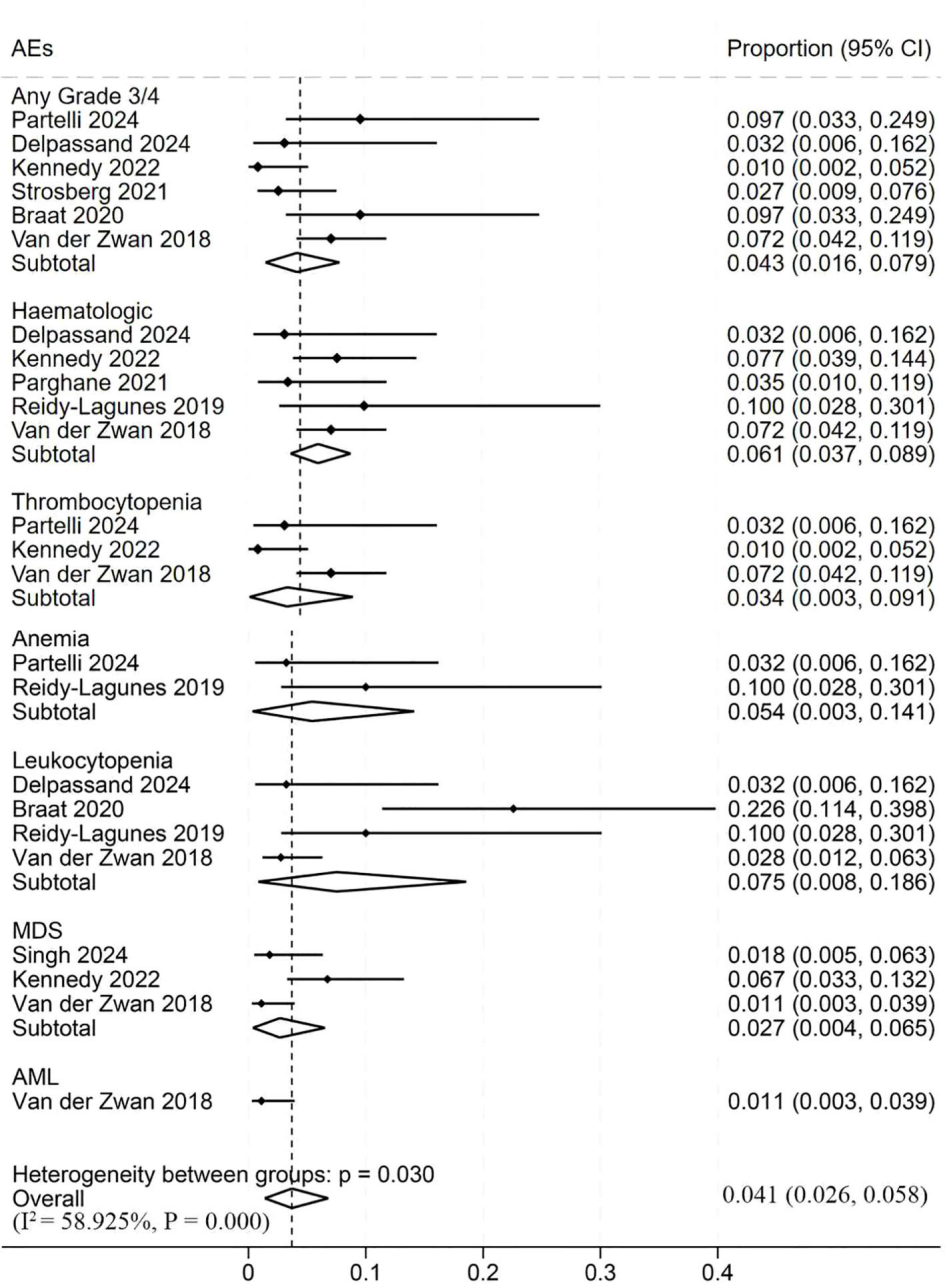
Forest plot of odds ratios comparing safety outcomes in patients treated with ^177^Lu-DOTATATE. The horizontal lines denote the upper and lower bounds of the 95% CI. *P*-values greater than 0.05 are considered to indicate statistical non-significance. ^177^Lu-DOTATATE -^177^Lu-DOTA-Tyr3-octreotate. MDS - Myelodysplastic syndrome. AML - Acute Myeloid Leukemia; CI - confidence intervals.

We also evaluated the safety profile of ^177^Lu-DOTATATE therapy. The adverse reactions were evaluated in 677 patients across 9 studies (**Figure 6**). The Cochran Q test indicated statistically significant heterogeneity in the overall incidence of adverse reactions across the included studies (*P* = 0.03), with an I² statistic of 58.925%, suggesting high heterogeneity. Thus, a random-effects model was applied for the meta-analysis. The overall incidence of adverse reactions was low 4.1% (95% CI, 2.6%–5.8%). The pooled incidence of grade ≥3 adverse reactions was 4.3% (95% CI, 1.6%–7.9%). Hematologic adverse events occurred in 6.1% of patients (95% CI, 3.7%–8.9%). Thrombocytopenia had a pooled incidence of 3.4% (95% CI, 0.3%–9.1%). Anemia was the most common adverse event 5.4% (95% CI, 0.3%–14.1%). Leukocytopenia occurred in 7.5% (95% CI, 0.8%–18.6%). Myelodysplastic syndrome (MDS) had an incidence of 2.7% (95% CI, 0.4%–6.5%). Acute Myeloid Leukemia (AML) is the least common adverse event. Among the 9 studies included, only one study from 2018 reported AML cases. The incidence rate was 1.1% (95% CI: 0.3%-3.9%) (27).

## 4 Discussion

In recent years, the incidence of NETs has steadily risen. Early clinical manifestations of NETs are often nonspecific, so most patients are diagnosed at an advanced stage (28). In this context, the limitations of traditional somatostatin analog (SSA) therapies have become increasingly apparent, underscoring a pressing need for more targeted novel treatments. ^177^Lu-DOTATATE achieves precise treatment by targeting somatostatin receptors overexpressed on NET cells, significantly improving patient survival outcomes.

However, despite growing clinical application, limited data are available regarding the efficacy of ^177^Lu-DOTATATE across the heterogeneous subpopulations of NETs, which vary considerably in tumor type, histological grade, and primary site. To address this knowledge gap, we conducted a comprehensive meta-analysis of 14 studies involving a total of 1844 patients to systematically evaluate the efficacy and safety of ^177^Lu-DOTATATE in the treatment of NETs. In terms of efficacy, the pooled DCR for ^177^Lu-DOTATATE treatment in NETs was 87.6%, and the pooled ORR was 32.2%. The pooled PFS across the included studies was 30.87 months, and the pooled OS was 51.85 months. These results support ^177^Lu-DOTATATE as an effective treatment option for NETs and demonstrate its clinical benefit. Regarding safety, our meta-analysis indicates that ^177^Lu-DOTATATE has a favorable profile with relatively few adverse reactions. Common side effects include hematologic toxicities such as anemia, thrombocytopenia, and leukocytopenia. In our data, grade ≥3 adverse events occurred in only 4.3% of patients treated with ^177^Lu-DOTATATE, compared to 14% with SSAs and 7% with everolimus, underscoring the superior safety of ^177^Lu-DOTATATE (29, 30).

In this study, DCR and ORR exhibited high heterogeneity (I² = 80.95% and 83.13%, respectively). This indicates substantial inter-study variability, potentially attributable to differences in NET primary sites and pathological grades. We therefore conducted a subgroup analysis by tumor grade. Patients with G1/G2 NETs demonstrated higher DCR and ORR than those with G3 NETs, suggesting that G1/G2 tumors derive greater benefit from PRRT. This disparity may reflect the lower malignancy and higher SSTR expression of G1/G2 tumors. Indeed, imaging shows G1/G2 NETs have greater ^68^Ga-DOTATATE PET uptake, indicating lower malignant potential. Conversely, G3 NETs exhibit lower ^68^Ga-DOTATATE PET uptake, consistent with their higher malignant potential (31). Additionally, G1/G2 NETs have a lower Ki-67 index, which may render them more sensitive to radionuclide therapy (32). Since PRRT efficacy depends on SSTR expression, the higher SSTR levels in G1/G2 tumors likely contribute to their superior outcomes (33, 34). Heterogeneity is also evident with respect to the primary tumor site. We compared PRRT efficacy across NETs of different origins and found that pancreatic NET patients had a markedly longer average PFS (93.9 vs 66.3 months for gastrointestinal NETs). One explanation is that pancreatic NETs generally express higher SSTR levels, allowing more effective targeting by ^177^Lu-DOTATATE (35). Additionally, 85% of pancreatic NETs are grade G1/G2, versus 78.6% of gastrointestinal NETs. Pancreatic NETs also tend to grow more slowly and have a better prognosis. These factors may account for the longer PFS observed in pancreatic NETs. Overall, these findings inform more precise selection of patients likely to benefit from PRRT.

Standardized PRRT using ^177^Lu-DOTATATE (7.4 GBq per infusion every 8 weeks for 4 cycles) remains the most widely used regimen. To further improve efficacy, two primary optimization strategies are suggested. First, a PRRT re-challenge (e.g., two additional cycles) is feasible for patients who show a favorable initial response and adequate tolerance, resulting in a substantial extension of PFS, with a reported increase of 9.6 months, while maintaining a comparable safety profile (17). Second, individualized treatment plans, particularly involving combination therapies, should be considered for specific patient subgroups. In cases of liver metastases, sequential PRRT in combination with locoregional therapies (such as transarterial chemoembolization, radiofrequency ablation, or transarterial embolization may enhance PFS and symptom control, particularly in PRRT sub-responders (36). Additionally, systemic combination approaches are under investigation. For instance, combining ^177^Lu-DOTATATE with metronomic capecitabine demonstrated a high DCR of 85% and a median PFS of 31.4 months (37). Importantly, the NETTER-2 study confirmed that ^177^Lu-DOTATATE plus octreotide LAR significantly prolonged PFS compared to octreotide LAR alone (22.8 vs. 8.5 months), thus establishing its therapeutic benefit.

Despite these positive findings, this meta-analysis has certain limitations. The included studies were predominantly cohort studies, with few high-quality RCTs, which introduces potential selection bias and other confounders. Additionally, unpublished negative results may not have been captured in our analysis. Furthermore, our analysis was essentially a single-arm study with high heterogeneity. On a positive note, we performed subgroup analyses by tumor grade and pathology for the first time, which may aid in developing more precise, individualized treatment plans.

## 5 Conclusions

This meta-analysis demonstrates that ^177^Lu-DOTATATE has a favorable efficacy and safety profile in patients with NETs. The findings further suggest that patients with well-differentiated (G1 or G2) NETs may derive particular benefit from ^177^Lu-DOTATATE therapy. Nevertheless, additional high-quality clinical trials, especially prospective randomized controlled trials, are warranted to confirm these conclusions and to facilitate the integration of ^177^Lu-DOTATATE therapy into clinical practice.

## Data Availability

The original contributions presented in the study are included in the article/**Supplementary Material**. Further inquiries can be directed to the corresponding authors.
