# Supplementary material for: 177Lu-labelled peptide receptor radionuclide therapy in patients with neuroendocrine tumors: a systematic review and meta-analysis
Source: Front Endocrinol (Lausanne). 2026 Feb 19;17:1758639. doi: 10.3389/fendo.2026.1758639 (PMC12960171; doi:10.3389/fendo.2026.1758639)
Supplement: Supplementary file 1 [file DataSheet1.docx]

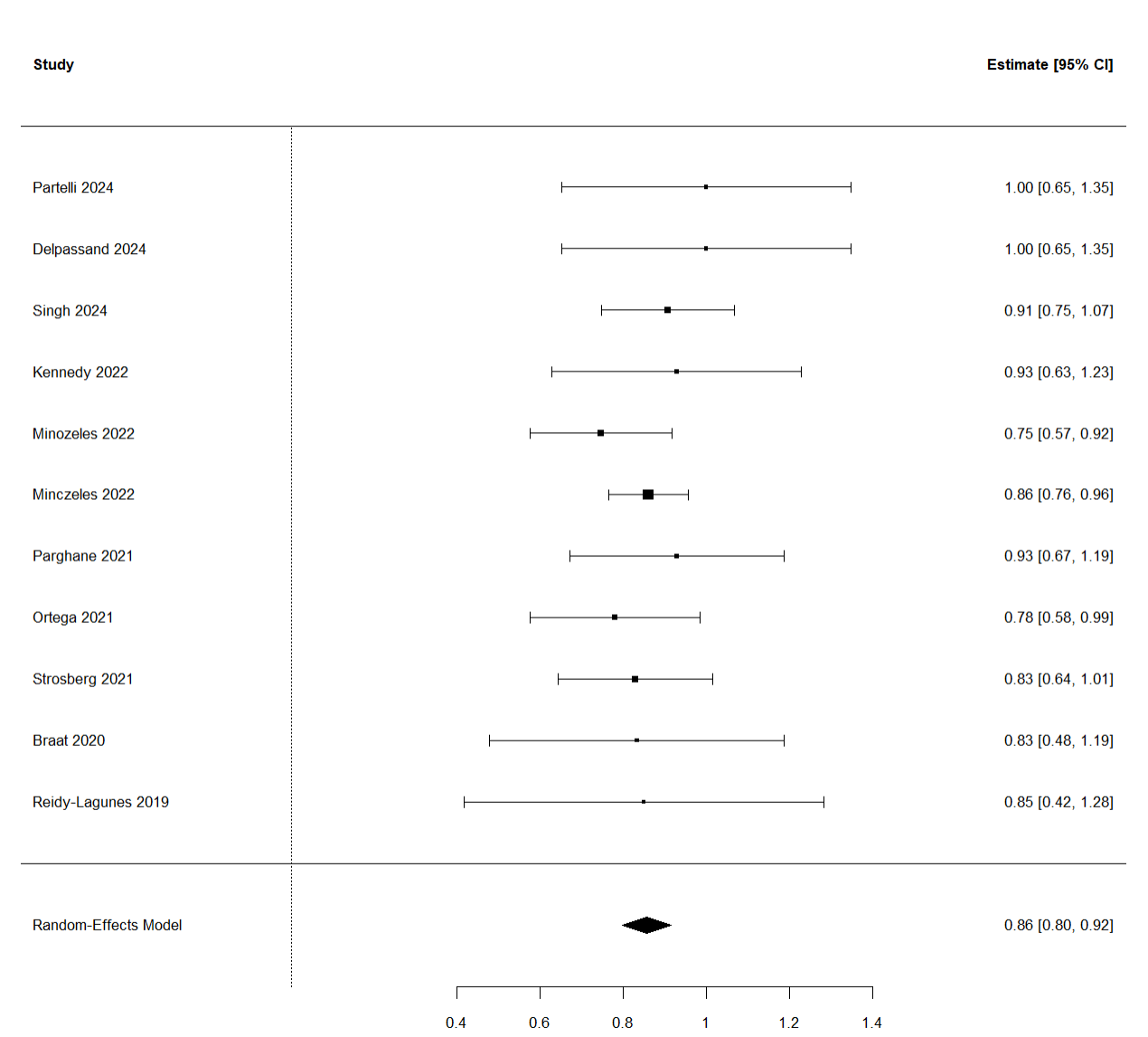


**Supplementary fig. 1.** Forest plot depicting the leave-one-out sensitivity analysis for DCR. DCR - disease control rate.


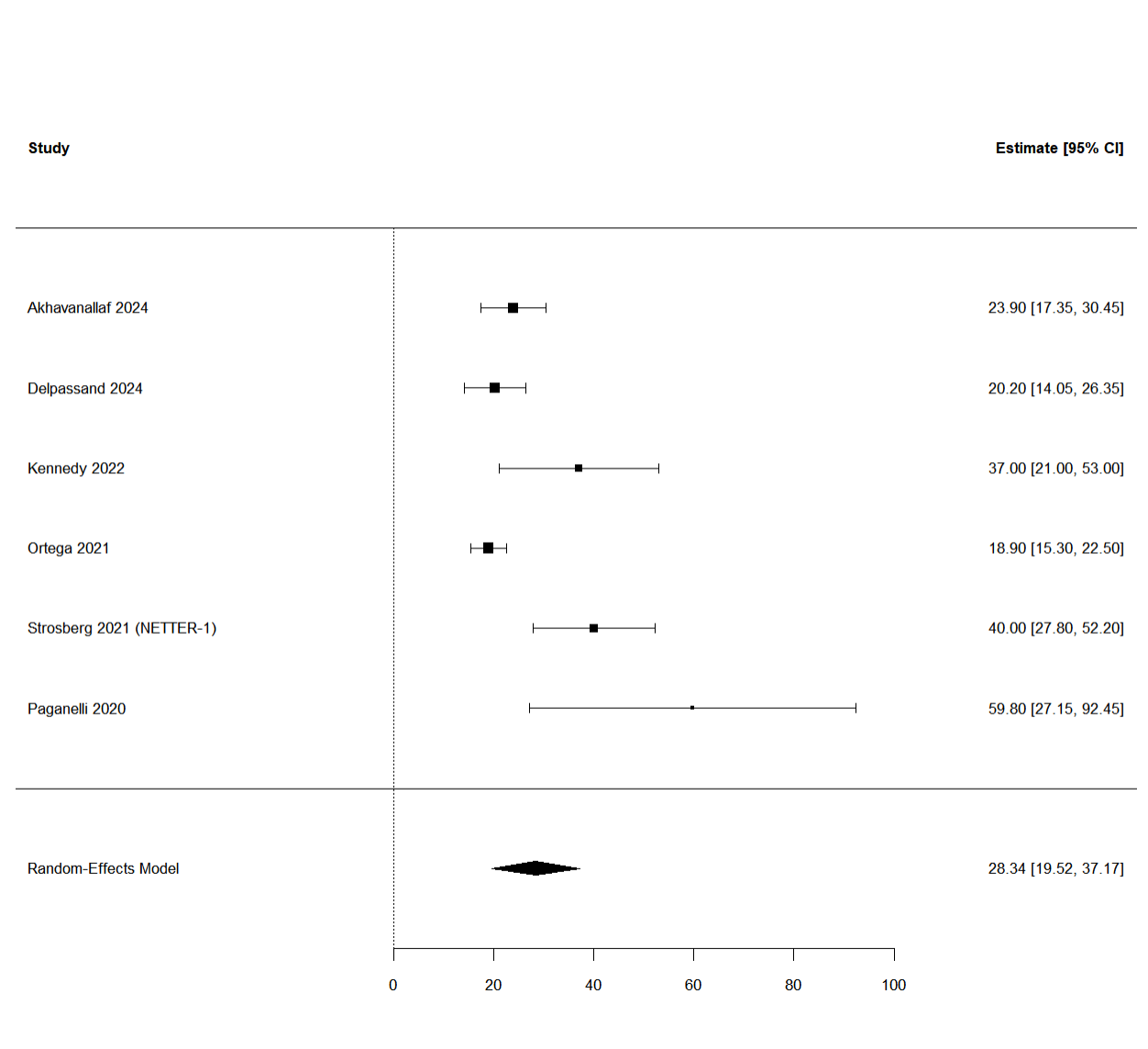


**Supplementary fig. 2.** Forest plot depicting the leave-one-out sensitivity analysis for PFS. PFS - progression free survival.
