# Supplementary material for: 177Lu-labelled peptide receptor radionuclide therapy in patients with neuroendocrine tumors: a systematic review and meta-analysis
Source: Front Endocrinol (Lausanne). 2026 Feb 19;17:1758639. doi: 10.3389/fendo.2026.1758639 (PMC12960171; doi:10.3389/fendo.2026.1758639)
Supplement: Supplementary file 2 [file Table1.docx]

**Supplementary table 1.** Primary data from leave-one-out sensitivity analysis of DCR. DCR - disease control rate.

| Study | ES | CL | UL | Z | I^2^ |
| --- | --- | --- | --- | --- | --- |
| Partelli 2024 | 0.8766 | 0.8055 | 0.9476 | 24.19 | 6.23 |
| Delpassand 2024 | 0.8766 | 0.8055 | 0.9476 | 24.19 | 6.23 |
| Singh 2024 | 0.8762 | 0.7964 | 0.9559 | 21.53 | 10.64 |
| Kennedy 2022 | 0.8787 | 0.8064 | 0.9510 | 23.83 | 7.41 |
| Minczeles 2022 | 0.9054 | 0.8335 | 0.9775 | 24.65 | 0 |
| Mitjavila 2022 | 0.8815 | 0.8123 | 0.9507 | 24.96 | 5.29 |
| Parghane 2021 | 0.8777 | 0.8043 | 0.9510 | 23.45 | 7.97 |
| Ortega 2021 | 0.8936 | 0.8205 | 0.9663 | 24.02 | 4.47 |
| Strosberg 2021 | 0.8891 | 0.8126 | 0.9653 | 22.82 | 8.41 |
| Braat 2020 | 0.8833 | 0.8119 | 0.9545 | 24.28 | 6.77 |
| Reidy-Lagunes 2019 | 0.8822 | 0.8116 | 0.9527 | 24.51 | 6.30 |
| van der Zwan 2018 | 0.8546 | 0.7808 | 0.9286 | 22.66 | 0 |

**Supplementary table 2.** Primary data from leave-one-out sensitivity analysis of PFS. PFS - progression free survival.

| Study | ES | CL | UL | Z | I^2^ |
| --- | --- | --- | --- | --- | --- |
| Akhavanallaf 2024 | 33.40 | 22.22 | 44.58 | 5.85 | 89.04 |
| Delpassand 2024 | 33.92 | 23.39 | 44.45 | 6.31 | 86.98 |
| Kennedy 2022 | 30.98 | 20.30 | 41.66 | 5.68 | 91.14 |
| Ortega 2021 | 34.16 | 24.06 | 44.25 | 6.63 | 81.92 |
| Strosberg 2021 | 30.18 | 19.78 | 40.59 | 5.68 | 90.04 |
| Braat 2020 | 29.62 | 20.55 | 38.69 | 6.39 | 88.79 |
| van der Zwan 2018 | 28.34 | 19.52 | 37.16 | 6.29 | 85.00 |
